# Optimization of L-Tryptophan Biosynthesis From L-Serine of Processed Iranian Beet and Cane Molasses and Indole by Induced *Escherichia coli* *ATCC* 11303 Cells

**DOI:** 10.5812/jjm.10589

**Published:** 2014-06-01

**Authors:** Tahereh Sadeghiyan-Rizi, Jamshid Fooladi, Majid Momhed Heravi, Sima Sadrai

**Affiliations:** 1 Department of Pharmaceutical Biotechnology, Faculty of Pharmacy, Isfahan University of Medical Sciences, Isfahan, IR Iran; 2 Department of Biology, National Laboratory of Industrial Microbiology, Faculty of Science, Alzahra University, Tehran, IR Iran; 3 Department of Chemistry, Faculty of Science, Alzahra University, Tehran, IR Iran; 4 Department of Pharmaceutics, Faculty of Pharmacy, Tehran University of Medical Sciences, Tehran, IR Iran

**Keywords:** Tryptophan, Tryptophan Synthase, Indole

## Abstract

**Background:**

L-tryptophan is an important ingredient in medicines, especially in neuromedicines such as antidepressants. Many commercial processes employ various microorganisms with high tryptophan synthase activity to produce L-tryptophan from indole and L-serine, but these processes are very costly due to the costs of precursors, especially L-serine.

**Objectives:**

For this reason, we studied the ability to use processed Iranian cane and beet molasses as L-serine sources for L-tryptophan production, which enables us to reach a cost-effective process.

**Materials and Methods:**

Whole cells of *Escherichia coli* *ATCC* 11303 were induced for L-tryptophan synthase by addition of indole to the growth medium and bacterial cells harvested from the growth medium were used as biocatalysts in the production medium. Conditions of the production medium were optimized and Iranian cane and beet molasses were processed by solvent extraction with ethanol and n-butanol and used as L-serine sources of the production medium. Amount of L-tryptophan and theoretical yield of L-tryptophan production were determined by High Performance Liquid Chromatography and by a colorimetrical method on the basis of the remaining indole assay, respectively.

**Results:**

L-tryptophan production increased by 15 folds, when indole was used as an inducer. L-tryptophan was produced from processed Iranian beet molasses in satisfactory amounts (0.53 mM) and no exogenous pyridoxal phosphate was required as a cofactor under our experimental conditions.

**Conclusions:**

The obtained results proved that Iranian beet molasses include significant amounts of L-serine that makes them a suitable substitution for L-serine. Findings of the present study give impetus to use of Iranian beet molasses for cost-effective L-Trp production in the amino acid industry.

## 1. Background

L-tryptophan (L-Trp) is a fundamental precursor of various neurotransmitters in the brain, which are essential for the regulation of mood, sleep, appetite and pain level, such as melatonin, niacin and serotonin (1-7). It is used widespread in the pharmaceutical industry for the chemical synthesis of a range of drugs such as antidepressants, sedative pharmaceuticals and drugs used for the treatment of schizophrenia and alcoholism (8, 9). It is also used as a food supplement in animal feeds (10). There are chemical and microbial processes for L-Trp production. All chemical synthesis yield DL-Tryptophan and the product must be resolved for the separation of the biologically active L-isomer (11). The majority of L-Trp production depends on microbial processes. These processes include direct fermentation from carbohydrates or hydrocarbons, enzymatic reaction from L-Trp precursors and bioconversion from L-Trp precursors. Effective production processes are available with mutants of *Escherichia coli* (12), *Corynebacterium glutamicum* (13), *Bacillus subtilis* (14) and *Brevibacterium lactofermentum* (15). 

Although, many industrial processes employ *E. coli* cells which have L-tryptophan synthase (TSase, EC 4.2.1.20) activity to convert indole and L-serine (L-Ser) to L-Trp (16); since this type of production is based on a very simple and one-step reaction therefore the complete biosynthetic pathway of L-Trp isn’t necessary and the complicated mutations of microorganisms which control the regulatory mechanisms are often not required (17). However, there are disadvantages to these processes because precursors are expensive. Indole is available from the petrochemical industry as a comparably inexpensive educt, whereas L-Ser is very expensive because a racemic mixture is formed during its manufacture. To address this problem, new methods for both process design and cheap precursor substitution have been developed, for example production of L-Ser from methanol and glycine by methanol-utilizing bacteria (17-19). Recovery of L-Ser from the by-products of various industries provides another means that may be more efficient than the latter.

## 2. Objectives

The present research aimed to bacterial cell propagation in the culture medium containing beet molasses as an inexpensive carbon source and indole as a Tryptophan Synthase inducer, optimization of production medium conditions and use of induced bacterial cells as biocatalysts of L-Trp production from indole and processed Iranian cane and beet molasses as the sole L-Ser source of production medium.

## 3. Materials and Methods

### 3.1. Chemicals

Indole, L-serine (L-Ser), pyridoxal phosphate (PLP), L-Trp, n-butanol and methanol were purchased from Merck (Germany) and all other chemicals were analytical grades or the highest purity commercially available. Cane and beet molasses were obtained from three Iranian sugar industries, cane and beet composition are presented in Table 1.

**Table 1.** Iranian Cane and Beet Molasses Composition ^a^

-	Polarity, %	Brix, %	Purity, %	pH
**Orumiyeh beet molasses**	46.2	77.3	59.77	6.4
**Karaj cane molasses**	36.8	62.5	58.88	7
**Fariman cane molasses**	34.6	60.3	57.38	7

^a^ Polarity = Sucrose content (g/100 mL molasses); Brix = total soluble solids (w/w); Purity% = (Polarity/Brix) × 100

### 3.2. Microorganism and Culture Conditions

*E. coli* *ATCC* 11303 was used in this research. The strain was maintained on an agar slant prepared from a complete medium containing glucose (20 g/L), yeast extract (25 g/L), ammonium sulphate (0.5 g/L) and agar (15 g/L). Bacterial colonies were inoculated in a 500 mL Erlenmeyer flask containing 100 mL of complete medium (as pre-culture) and incubated on a rotary shaker (180 rpm) at 37 ˚C for five hours. The cells recovered from 10 mL of pre culture medium were resuspended in 10 mL of 0.9% NaCl solution and 1 mL of this suspension was inoculated to a 500 mL Erlenmeyer flask containing 100 mL of growth medium composed of K_2_HPO_4_ (7 g/L), KH_2_PO_4_ (3 g/L), Na_3_.citrate.3H_2_O (0.5 g/L), MgSO_4_.7H_2_O (0.1 g/L) and beet molasses (16.2 g/L). After 6.5 hours of incubation in conditions mentioned above, indole (0.058 g/L) was added to the growth medium and incubation was continued for up to ten hours. The cells of stationary phase were harvested and used in L-Trp production medium as a biocatalyst. Polarity% of beet molasses (g sucrose/100 mL beet molasses) was 42.2%. Accordingly, 10.8 mL of beet molasses equal to 16.2 g, contained 5 g sucrose and was used as a carbon source for the culture medium.

### 3.3. Determination of Cell Growth

The growth of microorganisms was determined periodically by measuring the optical density at 620 nm, every one hour. For determination of biomass weight, culture medium was centrifuged and the harvested cell mass was washed twice and weighted (20).

### 3.4. Molasses Processing

Each of the two samples of cane and one sample of beet molasses (50 mL) were diluted by distilled water (1:1), mixed with ethanol 96% (1:1) and kept at 4˚C overnight, in order to precipitate macromolecules (such as protein, oligosaccharide, etc.) and dye. After solvent evaporation, the residue was mixed with n-butanol (1:1); the mixture was vigorously vortexed and incubated at room temperature for one hour. After formation of two phases, the organic phase was separated and n-butanol was evaporated. The residue from each molasses was used instead of L-Ser in L-Trp production medium.

### 3.5. Tryptophan Production

Biomass harvested from culture medium (3 g) was transferred to a 500 mL Erlenmeyer flask containing production medium (100 mL potassium phosphate buffer (0.1 M, pH = 8), indole (0.05 g) and L-Ser (0.05 g) incubated in a rotary shaker (180 rpm) at 37 ˚C. After four hours of incubation, the production medium was centrifuged at 12000 rpm for 20 minutes at 4˚C and the supernatant fluid was used for the assay of the produced L-Trp and the remaining indole.

L-Trp was detected by High Performance Liquid Chromatography in a Waters system equipped with an isocratic pump and an ultraviolet detector, by using an RP-18 column (MZ-analytical column, 4 × 100 mm) with 3 µm particle size; the mobile phase was phosphate buffer (0.05 M, pH = 4.2): methanol (70:30). Injection volume, flow rate and λ_absorbance_ were 50 µL, 1.2 ml/min and 220 nm, respectively (21, 22). For sample analysis, L-Trp was dissolved to a concentration of 1 mg/mL with purified water as stock solution. L-Trp concentrations of 5, 4, 2, 1, 0.5, 0.2, 0.1 and 0.05 µg/mL were prepared with purified water from stock solution and used for the standard curve. L-Trp concentration in the supernatant fluid of the production medium was determined according to the standard curve. AT 3000 Autochor software was used for HPLC data analysis. Consumed indole and the theoretical yield of L-Trp production was determined colorimetrically on the basis of the remaining indole in the supernatant fluid of production medium, determined spectrophotometrically at 490 nm (23).

### 3.6. Effect of Indole on Cell Growth and L-Trp Production

Indole has bacteriostatic effects on bacterial cells at specific concentrations (24). In this research indole presence in culture medium as the TSase inducer was investigated, thus it was a pre-requsite to determine the effect of indole on bacterial cells growth. *E. coli* *ATCC* 11303 was grown in the three culture media, one being without indole (control sample), and the two others with 0.058 g/L of indole; in one indole was added at the beginning (sample 1) and in the other it was added near to the end of the logarithmic phase, i.e. after 6.5 hours of growth medium incubation (sample 2). Next, 1 g of bacterial cells harvested from each of the three samples was transferred to the production medium containing 0.005 g of pyridoxal phasphate in addition to L-Ser and indole (0.05 g) and incubated for six hours. Cell growth was determined by measuring biomass weight during logarithmic, end of logarithmic and stationary phases in control samples and sample 1. Also L-Trp produced by biomass harvested from the three samples was compared to each other.

### 3.7. Effect of PLP, Time Course of Reaction and Biocatalyst Amount on L-Tryptophan Production

To establish the most advantageous production conditions, various parameters were optimized. TSase is a PLP-dependent enzyme (25). Exogenous PLP requirements as the cofactor of the reaction was investigated by addition of different PLP concentrations (0, 0.005, 0.01 and 0.02 g/100 mL) to the production medium and incubation for 6 hours, after which L-Trp produced by the 1 g biomass in the four production media were compared with each other. TSase and tryptophanase (TPase, EC 4.1.99.1) are both present in *E. coli*. TPase catalyzes the conversion of L-Trp to pyruvate, ammonia and indole while L-Trp concentration increases (26). To check when L-Trp degradation begins under our experimental conditions, production medium was incubated for 6 hours, sampling was done every one hour, produced L-Trp and remaining indole in each sample was assayed and optimum time course of the reaction was determined. The optimal amount of biocatalyst for L-Trp production was determined by the use of production medium containing different amounts of biocatalyst (1, 2, 3, 4 and 5 g/100 mL) incubated for four hours. Optimal biocatalyst amount was appointed by comparison of L-Trp production and indole consumption among these samples.

### 3.8. Investigation of L-Trp Production by Processed Iranian Beet and Cane Molasses

After optimization of tryptophan production conditions, Iranian cane and beet molasses capability to be used as an L-Ser source of the production medium was investigated. L-Ser of the production medium was displaced with processed Orumiyeh (northwest of Iran) beet molasses and Karaj (center of Iran) and Fariman (northeast of Iran) cane molasses.

Since other components except L-Ser might exist in processed molasses and affect the reaction, the production medium was incubated for a longer duration i.e. 10 hours and sampling was done at four, eight and ten hours. Since processed molasses might contain L-Trp and interfere in the results, L-Trp existing in the production medium was calculated before incubation (T0).

### 3.9. Statistical Method

The experiments were carried out in triplicates. All the results were analyzed statistically by one-way analysis of variance and Tukey’s test with 95% confidence level using Minitab 15.2.

## 4. Results

### 4.1. Effect of Indole on Cell Growth and L-Trp Production

**Figure 1.**
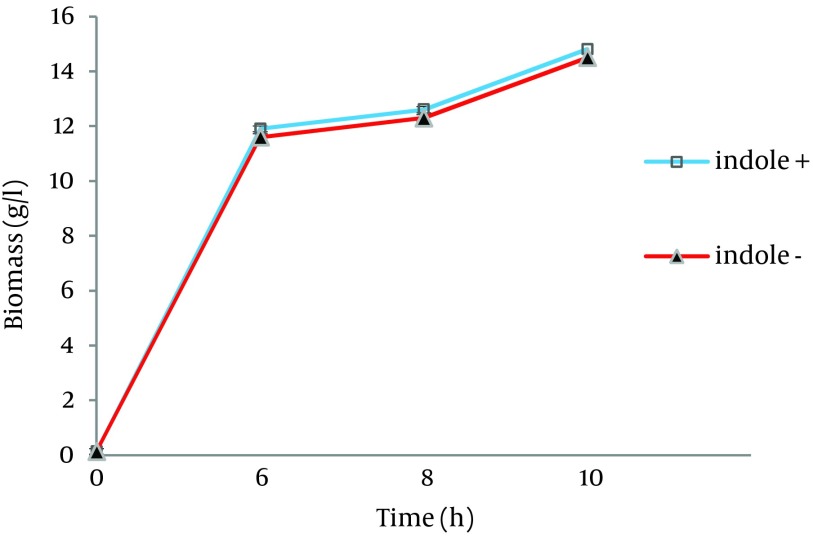
Effect of Indole on Growth of *E. coli* *ATCC* 11303 The Organism was grown at 37 ˚C and 180 rpm in the following media: ▲ Growth medium containing K_2_HPO_4_ (7 g/L), KH_2_PO_4_ (3 g/L), Na_3_.citrate.3H_2_O (0.5 g/L), MgSO_4_.7H_2_O (0.1 g/L) and beet molasses (16.2 g/L) (control sample); □ Growth medium containing K_2_HPO_4_ (7 g/L), KH_2_PO_4_ (3 g/L), Na_3_.citrate.3H_2_O (0.5 g/L), MgSO_4_.7H_2_O (0.1 g/L), beet molasses (16.2 g/L) and 0.058 g/L indole (sample 1).

**Figure 2.**
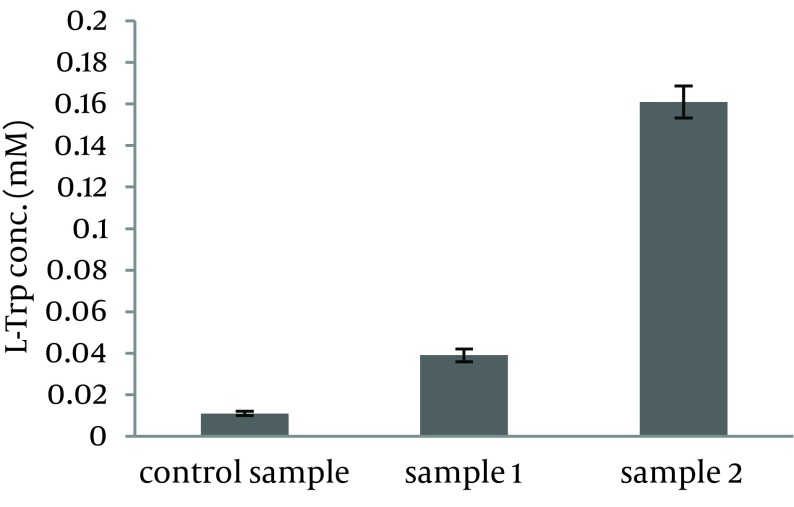
Effect of Indole on L-Trp Production Biomass harvested from the growth medium containing K_2_HPO_4_ 7 g/L, KH_2_PO_4_ 3 g/L, Na_3_.citrate.3H_2_O 0.5 g/L, MgSO_4_.7H_2_O 0.1 g/L and beet molasses 16.2 g/L (control sample) was transferred to the production medium (1); Biomass harvested from the growth medium containing K_2_HPO_4_ 7 g/L, KH_2_PO_4_ 3 g/L, Na_3_.citrate.3H_2_O 0.5 g/L, MgSO_4_.7H_2_O 0.1 g/L, beet molasses 16.2 g/L and indole 0.058 g/L (sample 1) was transferred to the production medium (2); Biomass harvested from the growth medium containing K_2_HPO_4_ 7 g/L, KH_2_PO_4_ 3 g/L, Na_3_.citrate.3H_2_O 0.5 g/L, MgSO_4_.7H_2_O 0.1 g/L, beet molasses 16.2 g/L and indole 0.058 g/L (that was added to it after 6.5 hours of growth medium incubation (sample 2), was transferred to the production medium (3) and incubated at 37˚C and 180 rpm for six hours.

As shown in Figure 1, bacterial growth was not affected by indole addition to the growth culture. Results showed that L-Trp produced by the biocatalyst, grown in sample 1 and sample 2, was increased by 3.7 and 15 folds, respectively (Figure 2). Therefore, indole addition to the growth medium was carried out 6.5 hours after growth medium incubation for subsequent experiments.

### 4.2. Effect of PLP, Time Course of Reaction and Biocatalyst Amount on L-Trp Production 

**Figure 3.**
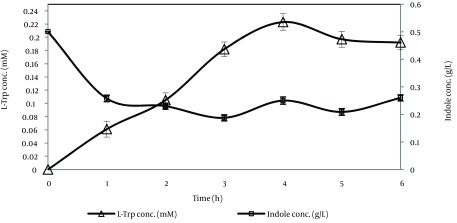
Time Course of L-Trp Production and Indole Consumption Time Course of L-Trp Production (∆) and Indole Consumption by *E. coli* *ATCC* 11303 in a Production Medium Containing L-Ser and indole (0.5 g/L) Incubated at 37˚C and 180 rpm for six Hours.

**Figure 4.**
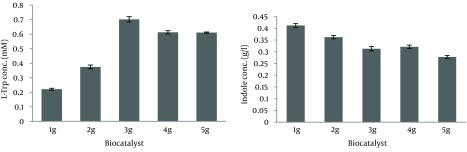
Effect of Biocatalyst Amount on L-Trp Production and Indole Consumption Effect of Biocatalyst Amount on L-Trp Production (A) and Indole Consumption (B) by Biomass (1-5 g/100 mL) of *E. coli* *ATCC* 11303 in the Production Medium Containing L-Ser and Indole (0.5 g/L) Incubated at 37 ˚C and 180 rpm for Four Hours.

It was indicated that no exogenous PLP was needed under our experimental conditions (Table 2). As shown in Figure 3, L-Trp production increased for up to four hours and this period, it decreased due to degradation by TPase. On the other hand, the remaining indole gradually decreased for up to three hours and after this period it increased, therefore the best time course for L-Trp production was four hours and L-Trp production was stopped at four hours in subsequent tests. As shown in Figure 4, most of the L-Trp production was obtained by use of 3 g of biomass. After optimization of production parameters, theoretical yield of L-Trp production reached 37.4%.

**Table 2.** Effect of PLP on L-Trp Production ^a^

Concentration of PLP, g/L	L-Trp Concentration, mM
**None**	0.187
**0.05**	0.158
**0.1**	0.103
**0.2**	0.064

^a^ Abbreviations: L-Trp, L-tryptophan; PLP, pyridoxal phosphate.

### 4.3. Investigation of Processed Molasses Usage Instead of L-Ser

L-Trp was produced from processed Orumiyeh beet molasses as the L-Ser source of reaction; whereas processed cane molasses didn’t contain L-Ser and L-Trp was not produced by use of them as the L-Ser source in the reaction mixture (Table 3).

**Table 3.** L-Trp Production in the Presence of Processed Molasses as L-Ser Source

L-Ser Source of Production Medium	L-Trp Concentration, mM			
	0 Hour ^a^	4 Hours	8 Hours	10 Hours
**Orumiyeh beet molasses**	0.025	0.173	0.53	0.447
**Fariman cane molasses**	0.0007	0.0004	0	0
**Karaj cane molasses**	0	0	0	0
**L-Ser**	0	0.702	-	-

^a^ Existing L-Trp in processed molasses.

## 5. Discussion

The cost-effective production of L-Trp has gradually attracted the attention of the amino acid industry (11). In our previous work, cane molasses (27) and combination of cane and beet molasses accompanied by 0.35 g/L L-Ser (28) were used as L-Ser sources in the production medium. In the present study, L-Trp production was improved by optimization of culture and production medium conditions and processing of beet molasses before use as the sole L-Ser source, consequently L-Trp production reached 0.53 mM (Table 3).

Beet molasses was used not only as the L-Ser source in the production medium but also as the carbon source in the culture medium. Beet molasses contains relatively large amounts of sucrose, amino acids and vitamins such as pyridoxine (29). For this reason, in our study use of beet molasses as the carbon source of culture medium not only accelerated the bacterial cells growth but also these cells gained a significant amount of PLP from the culture medium and when used as a biocatalyst in the production medium, they didn’t require exogenous PLP as a cofactor of TSase (Table 2) whereas in previous reports exogenous PLP was needed (10, 11, 13, 16, 17, 30-32); this finding is very important from an economical point of view.

In this research, inducer effect of indole was proved by the fifteen-fold increase in L-Trp production. Also it was found that indole addition near to the end of the logarithmic phase was more effective because L-Trp as well as other amino acids is produced in the stationary phase (Figure 2). Langrene et al. (33) used three-indolacrylic acid and observed an increased L-Trp production by ten folds. As the main result, L-Trp was produced from processed Orumiyeh beet molasses, as the L-Ser source. Although, it was observed that use of processed beet molasses as the L-Ser source of production medium leads to a 17.2% decrease in L-Trp production compared to when L-Ser was used (Table 3). This was expected, since processed beet molasses contain considerable amounts of L-Trp (0.025 mM in the reaction mixture, on the other hand 1.02 mg/50 mL molasses) (Table 3) and L-Trp is a strong inhibitor of L-Trp production.

In a report by Mateus et al. (9) an initial L-Trp led to a decrease in its initial production rate. The two samples of cane molasses used in this study didn’t have enough L-Ser, and L-Trp was not produced in their presence (Table 3), whereas in a previous study (27) L-Trp was produced from the L-Ser content of cane molasses. The samples of cane molasses used in the two studies were prepared from different sugar factories and it has been proved that composition of molasses is influenced by factors such as soil type, ambient temperature, moisture, variety, production practices at a particular processing plant and storage variables (34, 35). In this research, we proved that Iranian beet molasses includes significant amounts of L-Ser that makes it a suitable substitution for L-Ser in L-Trp production. Findings of the present study give impetus to the use of Iranian beet molasses for cost-effective L-Trp production on an industrial scale. However, further experimentation to obtain more pure L-Ser fractions from Iranian molasses and consequently more production of L-Trp promises to shed more light on this subject and our future work will be aimed at recovery of L-Ser from beet molasses using ion-exclusion and ion-exchange chromatography.
